# A Survey of Soil Enzyme Activities along Major Roads in Beijing: The Implications for Traffic Corridor Green Space Management

**DOI:** 10.3390/ijerph121012475

**Published:** 2015-10-08

**Authors:** Tianxin Li, Linglong Meng, Uwizeyimana Herman, Zhongming Lu, John Crittenden

**Affiliations:** 1 School of Civil and Environmental Engineering, University of Science and Technology Beijing, Beijing 100083, China; E-Mails: linglongmeng2013@126.com (L.M.); uwiherman05@yahoo.fr (U.H.); 2 The State Key Laboratory of Water Environment Simulation, School of Environment, Beijing Normal University, Beijing 100875, China; 3 School of Civil and Environmental Engineering, Georgia Institute of Technology, Atlanta, GA 30332, USA; E-Mails: Zhongming.Lu@gatech.edu (Z.L.); john.crittenden@ce.gatech.edu (J.C.); 4 Brook Byers Institute for Sustainable Systems, Georgia Institute of Technology, 828 West Peachtree Street, Atlanta, GA 30332, USA

**Keywords:** soil enzymes, traffic pollutants, green space management, soil degradation, Beijing

## Abstract

Soil quality is critical to the management of urban green space, in particular, along traffic corridors where traffic-related air pollution is significant. Soil quality can be evaluated by soil enzyme activities, which show quick responses to both natural and anthropogenic disturbances. In this study, we investigated three soil enzyme activities (*i.e.*, dehydrogenase, catalase and urease) along the major roads in urban areas of Beijing. Results show the activities of dehydrogenase, catalase and urease in urban samples were 58.8%, 68.2% and 48.5% less than the rural sample, respectively. The content of fluorescent amino acids as indicators of microbial activities was also consistently lower in urban samples than the rural. We observed two times greater exposure of particulate material along the roadsides in urban areas than rural areas. Although traffic air pollutants provide some nutrient sources to stimulate the URE activity, the exposure to traffic-related air pollution leads to the substantial decrease in enzyme activities. There were significant negative correlations for exposure to PM10 with DHA (*r* = −0.8267, *p* = 0.0017) and CAT (*r* = −0.89, *p* = 0.0002) activities. For the urban soils URE activity increased with the increasing of PM. We conclude that the degraded soil quality can negatively affect the target of developing plants and green spaces along the traffic corridors to mitigate the traffic impact. This study suggests the investigation of integrated strategies to restore the soil quality, reinforce the ecological service functions of green spaces along the traffic corridors and reduce the traffic pollutants.

## 1. Introduction

Urban green space management plays an important role in mitigating the impact of human activities and maintaining the ecosystem services of cities. The importance of this role is more significant along the roads because of traffic-related air pollution. For example, plants along the major roads can reduce the heat island effect [1] and reduce the diffusion of exhaust emissions [2]. Green spaces can treat the storm water runoff from the impermeable road surfaces and improve the quality of storm water runoff [3]. However, the successful development of green spaces along the traffic corridors requires high quality soil. Degradation of soil quality can impede the development of green spaces. The benefits of green spaces might not be significant when soil quality is not well maintained. For example, the installation of green infrastructures (e.g., bioswales, bioretention basins) may not work as an effective storm water management strategy where soil quality for vegetation is low [4]. Therefore, the degradation of soil quality along the traffic corridors and the impact of human activities should be assessed in order to develop cost-effective solutions for green space development.

Soil enzymes play essential roles in nutrient cycling and energy transformation by catalyzing numerous chemical, physical and biological reactions [5]. These reactions include oxidation, reduction, and hydrolysis. Soil enzymes are responsible for converting organic substances into nutrients to stimulate plant growth and maintain the environmental conditions (e.g., pH, electronic potential, and alkalinity) in the soil. Soil enzyme activities are very sensitive to environmental pollution [6]. Previous studies have suggested that soil enzymes can be potential indicators of soil quality and health [7,8]. Soil enzyme activities have also been used to evaluate soils with the heavy metal contamination [9] and to monitor the changes in agricultural ecosystems [10].

Vehicle exhaust emissions are major pollutants in urban areas. The rapid increase in vehicle usage for daily transportation in developing countries has caused an increase of urban environmental pollution. Mobile sources contribute to the emission of major urban air pollutants including: carbon monoxide (CO), nitrogen oxides (NO_x_), sulfur oxides (SO_2_), particulate matter (PM), heavy metals (Cd, Cu, Pb, Zn, *etc.*), volatile organic compounds (VOC_s_) and Ozone (O_3_) [11]. Roadside soils show a high degree of contamination from motor vehicles [12,13]. The concentration of heavy metals in the soils and plant tissues were high in the areas of higher traffic density [13]. High concentrations of heavy metals can reduce the rates of soil respiration, nitrogen mineralization and soil enzyme activities [14]. For example, the presentence of copper can inhibit the activity of soil urease and further limit nutrient availability to plants [15].

As a developing country, China will continue to urbanize for the next 20 years. Over 350 million Chinese city dwellers will add to the urban population by 2025. To maximize the role of green spaces in mitigating human impacts, we need to address the degradation of soil quality caused by the traffic-related pollutants. As an initial effort, we investigated the soil enzyme activities in ten roadsides in urban areas of Beijing and one roadside in rural area of Yanqing, Beijing. We compared both the loss of soil enzyme activities along the roadside and the exposure to traffic-related air pollutants between the urban and rural areas. In the discussion, we propose a hypothetical pathway model to explain how traffic-related air pollutants affect the soil enzyme activities. Finally, we propose a set of technical solutions to improve soil quality and reduce the traffic pollution.

## 2. Experimental Section

### 2.1. Soil Collection and Sampling

**Figure 1:**
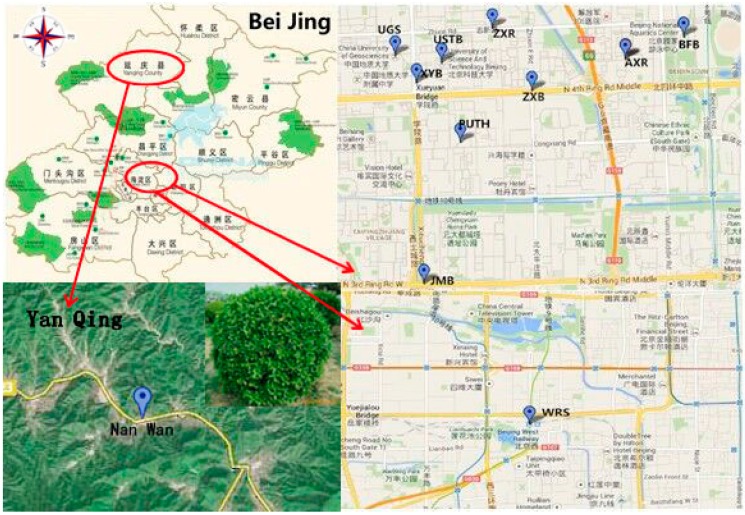
Locations of samples.

Soil samples were collected from the roadsides of ten different cross-roads with heavy vehicular flow in urban areas of Beijing and one control sample in rural areas of Beijing (Figure 1).

The sampling sites were: University of Science and Technology Beijing (USTB), West Railway Station (WRS), Peking University Third Hospital (PUTH), Anxiang Road (AXR), Zhixin Road (ZXR), Beijing Foreign Bureau (BFB), Xueyuan bridge (XYB), University of Geosciences (UGS), Jimen bridge (JMB), Zhixin bridge (ZXB), and Yanqing (YQ) as the control sample (see Supplementary Information for pictures). Each site was divided into three representative blocks and during each sampling five soil samples were collected randomly from 0 cm to 15 cm soil depth. The soil was air-dried and screened through 2 mm sieve to eliminate plant and animal residues. The sample was preserved at 4 °C for enzyme activities analysis. We measured basic soil properties (organic matters, and total nitrogen) in this study.

### 2.2. Measurement of Enzyme Activities

Dehydrogenase (DHA) activity was measured using the classical triphenyl tetrazolium chloride method [16]. In a test-tube, 5 g of sieved soil, 0.4 g of CaCO_3_, 1 mL of 1.5% 2,3,5- triphenyltetrazolium chloride and 2.5 mL of pure water were added and well mixed. The tubes were sealed and incubated for 24 h at 37 °C in the dark. The product 1,3,5-triphenylformazan (TPF), from the reduction of triphenyltetrazolium chloride, was extracted using methanol and additional methanol was added to make the sample volume of 50 mL. The 1,3,5-triphenylformazan concentration was determined by the spectrophotometric method at 485 nm and methanol was used in the reference cell. DHA activity was expressed as µg TPF/g/h.

Catalase activity was determined by potassium permanganate titration [17]. We added 5 g soil and 0.5 mL of toluene into a triangular flask. The mixed solution was kept in the refrigerator at 4 °C for 30 min. Next, we added 5 mL of 3% H_2_O_2_ solution to the mixed solution and kept the solution in the refrigerator for another 1 h. Then, we added 2 mol/L of H_2_SO_4_ to the mixed solution. The filtrate was titrated with 0.01 mol/L of KMnO_4_ until the end-point of the faint pink colored solution. CAT activity was expressed in mL 0.1 mol/L KMnO_4_ solution titrated/(g dry soil 20 min).

Urease activity was determined using urea as the substrate [18]. The soil mixture (5 g soil, 1 mL toluene, 10 mL of 10% urea solution, and 20 mL of citrate buffer pH 6.7) was incubated at 37 °C for 24 h. We added 4 mL sodium phenolate (12.5% (w/v) phenol + 5.4% (w/v) NaOH) and 3 mL of 0.9% sodium hypochlorite to the filtrate, whereupon a red color appeared. The released NH_3_-N was determined spectrophotometric ally at 578 nm. The URE release rate was expressed as μg NH_3_-N/g/h.

Fluorescence spectroscopy is an effective method to investigate the molecular conformation of proteins. The proteins are excited to their electronic vibrational states by absorbing a photon. The proteins drop down to the ground electronic state by emitting the light [19]. By analyzing the frequencies and the relative intensities of the light emitted, we can determine the concentration of active proteins. The higher intensity of the lights emitted corresponds to the higher concentration of active proteins. Soil samples collected around roadside areas in Beijing were dried in the oven at the temperature of 105 °C. We added 1 g of each soil sample and 99 mL sterile water into 150 mL conical flasks. The solutions were mixed for 20~30 min at 25 °C. The supernatant of fermentation broth was collected and diluted 100-fold. The diluents were filtered through 0.45 μm glass fiber filter membrane before entering the fluorescence analyzer (Cary Eclipse, Varian Science and Technology Co., Ltd., CA, USA). The excitation light source was a xenon lamp. We collected the intensity of emission light ranging from 220 nm to 300 nm. The scanning rate was 1200 nm/min, and the ambient temperature was 24 °C.

### 2.3. Traffic-Related Air Pollution

PM_2.5_ (PM with aerodynamic diameter of less than 2.5 µm) and PM_10_ (PM with aerodynamic diameter of less than 10 µm) were adopted as indicators of traffic-related air pollution. Previous study shows that vehicle emissions is the major source of roadside PM in Beijing [20]. In January, the air pollution is typically much heavier than that in March. The concentrations of PM_10_ and PM_2.5_ were measured on hazy days in January and clear days in March. For comparison, we collected the PM_2.5_ and PM_10_ for two days in both the January and March, 2013, respectively. The PM_2.5_ and PM_10_ were measured by a DUSTTRAK II Aerosol Monitor (TSI Instrument Co., Ltd., Shoreview, MN, USA). The air was collected 2 m above the ground and within a circle of 2 m around the sampling points. We assumed the major contribution to the PM_2.5_ and PM_10_ was from on-road vehicle emissions and short-range transport. Real time measurements were carried out continuously for 12 h per day from 08:00 AM to 08:00 PM and 6 measurements were made each hour. Zeroing of the instruments was carried out automatically before each measurement.

## 3. Statistical Analysis

Statistical analyses were performed using SPSS Statistics 18 software (SPSS Inc., Chicago, IL, USA). The data from soil analyses were subjected to a two tailed *t*-test with a significance level of *p* < 0.05 to test the significance of soil physical-chemical properties, soil enzyme activities and traffic pollution among urban and rural soil samples.

**Table 1:** Physical and chemical parameters in rural and urban soils of Beijing.

Sample	Bulk Density (g/cm^3)^	Porosity %	Permeability mm/min	pH	Organic Matter (mg/kg)	Soil Nutrients	
						Total Nitrogen (mg/kg)	Available Phosphorus (mg/kg)
WRS	1.28	49.66	20.35	8.21	10.97	0.62	16.82
PUTH	1.47	46.52	10.34	7.44	13.14	0.71	17.34
AXR	1.35	48.26	18.96	8.35	10.05	0.85	16.53
ZXR	1.52	45.39	8.93	7.82	14.27	0.67	18.68
BFB	1.24	52.15	21.37	8.26	9.75	0.75	14.60
XYB	1.44	47.36	10.74	8.38	13.46	0.56	20.46
UGS	1.18	54.38	12.36	7.67	10.53	0.92	18.61
JMB	1.21	52.75	29.26	8.29	11.52	0.65	21.69
ZXB	1.38	46.53	18.62	8.32	13.61	0.78	17.73
USTB	1.26	50.48	25.41	8.16	10.44	0.69	18.37
YQ	1.15	57.86	31.69	6.89	32.27	1.06	8.32

## 4. Results and Discussion

### 4.1. Physical and Chemical Parameters

In the present study we also measured physical and chemical parameters of urban and rural soils (bulk density, porosity, permeability, pH, organic matter, total nitrogen, phosphorus; Table 1). Previous investigation on soil property in Beijing metropolitan region shows no significant differences in soil pH, bulk density, and total nitrogen (TN) between rural and urban areas [21]. Our results were consistent with previous research findings. There was no significant difference in the content of rural and urban soil parameters.

### 4.2. Dehydrogenase, Catalase and Urease Activities

**Figure 2:**
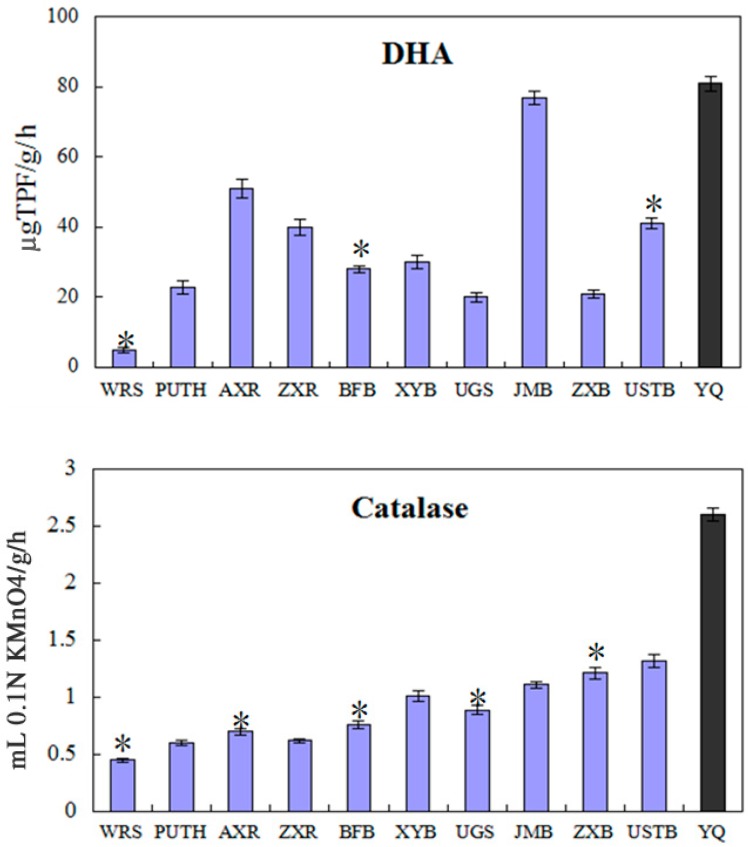

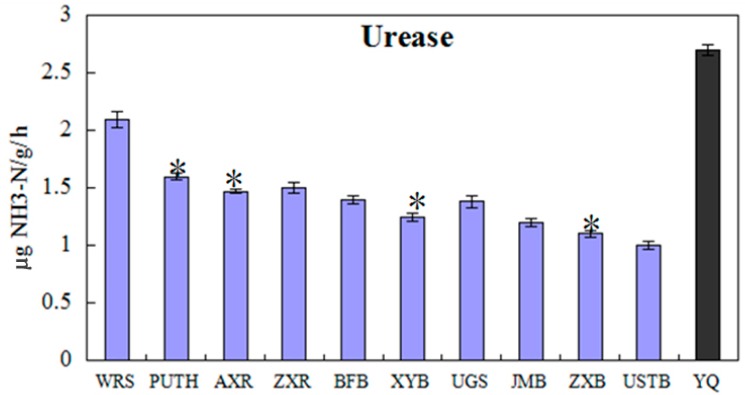
The dehydrogenase, catalase and urease activities in the study samples (light dark) and control sample (dark). Error bars are S.E; * indicates variance yields.

The soil enzyme activities of rural and urban samples are shown in Figure 2. The DHA of the urban samples varies from 6.57 µg TPF/g/h to 82.52 µg TPF/g/h. The data revealed that DHA was statistically lower in the urban samples that the rural sample. On average, the DHA of the roadside soil in urban area of Beijing is 58.8% lower than that of the rural sample. The catalase activity of the urban samples shows a range of 0.40 to 1.30 mL 0.1 N KMnO_4_/g /h. The comparison between rural and urban samples shows that the average catalase activity of the roadside soil in urban areas of Beijing is 68% less than that of the rural sample 2.64 mL 0.1 N KMnO_4_/g /h).

The urease activity of the urban soil samples showed a range of 1.05 to 2.11 μg NH_3_-N/g/h. Similar to the results of dehydrogenase and catalase, the rural sample has a higher urease activity (2.70 μg NH_3_-N/g/h). 

### 4.3. Fluorescence Activity Analysis

**Figure 3:**
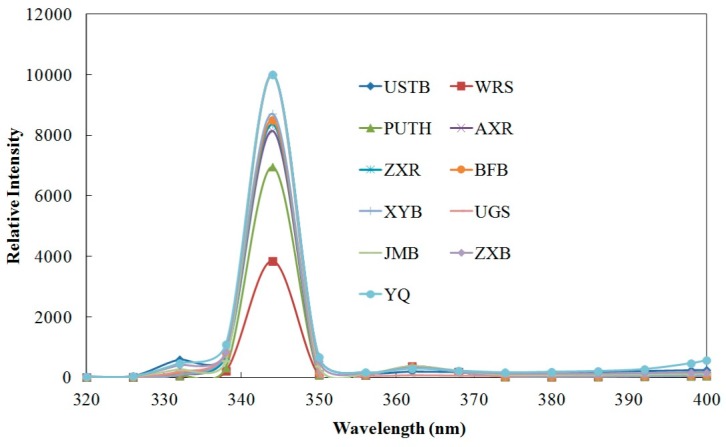
Result of fluorescence spectroscopy: higher relative intensity represents higher concentration of microbial activities.

Fluorescence spectroscopy was used to evaluate the influence of traffic pollution on soil enzyme activities (Figure 3). The fluorescence in natural environment is attributed mostly to humic substances derived from the breakdown of plant material and proteins produced through microbial activity. The fluorescent amino acids denoting the presence of proteins in soil are tryptophan, tyrosine and phenylalanine. The fluorescence intensity of these compounds can be used as indicators. 

Indicators of microbial activity in soil and can give image of the soil ecosystem health [22,23]. Protein-fluorescence peaks were observed at 340–350 nm. The same peak means that the content of active amino acids is close among the soil samples. The similarity of active protein composition implies the background of soil property is close among the soil samples in Beijing. The relative intensity of light emitted is higher for the rural sample soil than the ten samples in urban areas of Beijing. The higher fluorescence intensity shows the higher amino acids abundances in the rural sample. In contrast, the fluorescence intensity is lower in the ten samples along the major roads in urban areas of Beijing.

### 4.4. Traffic-Related Air Pollutions

In the January, the average PM_2.5_ and PM_10_ concentrations in the ten urban sample sites were 231.95 µg/m^3^ and 260.95 µg/m^3^. In contrast, the PM_2.5_ and PM_10_ concentration in the rural sample in Yanqing was 77.5 µg/m^3^ and 85.5 µg/m^3^. In March, the average PM_2.5_ and PM_10_ concentration in the ten urban sample sites was 52.45 µg/m^3^. The PM_2.5_ and PM_10_ concentration was 20.5 µg/m^3^ in the rural sample in Yanqing. On average, the PM_2.5_ and PM_10_ concentrations along the roadsides in urban areas were 2–3 times higher than those in the rural sample in Yanqing. 

### 4.5. The Impact of Traffic-Related Air Pollution on Soil Enzyme Activities

The factors that can affect the soil enzyme activities include natural factors (moisture, oxygen, plants, pH, *etc.*), and anthropogenic factors (Co, heavy metals, ozone, *etc.*). In this study, the soil in the ten urban sample sites and the rural control sample site is covered by the same plant, *Buxus megistophylla*. We found that soil enzyme activities were lower where increases in haze day PM2.5 and PM10 were higher (Table 2). Urban PM2.5 and PM10 were 3.15- and 3.24-fold higher, respectively, than rural PM concentrations, while urban DHA, CAT, and URE activities were 41%, 32%, and 51% of respective rural soil activities.

**Figure 4:**
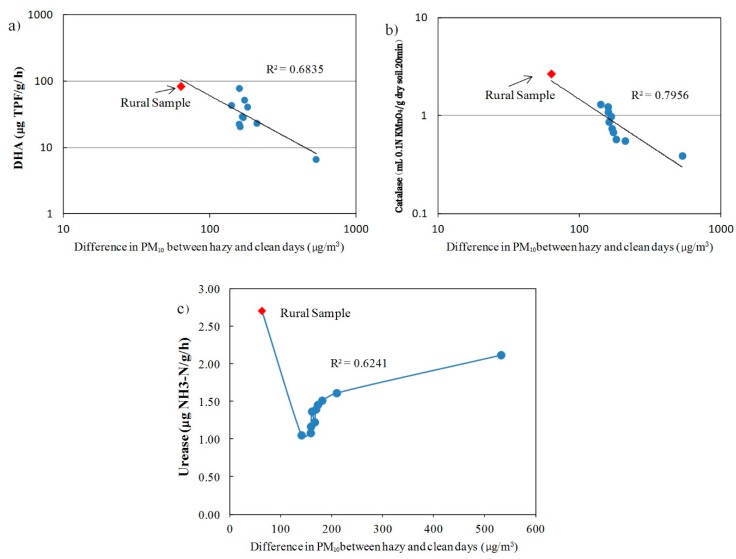
The impacts of exposure to PM_10_ on soil enzyme activities: (**a**) DHA; (**b**) catalase and (**c**) urease.

We further investigated the relationships between the exposure to air pollution (PM10) and soil enzyme activities for individual sites (Figure 4). There were significant negative correlations for exposure to PM10 with DHA (*r* = −0.8267, *p* = 0.0017) and CAT (*r* = −0.89, *p* = 0.0002) activities. In contrast, urban soil URE activities increased with increasing PM exposure while remaining substantially lower than activity in the rural sample where the PM exposure was much lower.

**Table 2:** The comparison of pollutant exposure and soil enzyme activities between the rural sample in Yanqing and the average of the ten urban samples in urban areas of Beijing.

Category	YQ	Average of the Ten Urban Sample Sites
The increase in PM_10_ exposure during the haze day (µg/m^3^)	63.5	205.5
The increase in PM_2.5_ exposure during the haze day (µg/m^3^)	57.0	179.5
DHA (µg TPF/g/h)	82.52	34.00
Catalase (mL 0.1 N KMnO_4_/g /h)	2.64	0.84
Urease (µgNH_3_-N/g/h)	2.70	1.39

### 4.6. Enzyme Activities and Traffic Pollution

The analysis of soil enzymatic activities showed the lower soil enzyme activities in the soil along the major roads in urban areas of Beijing than that in the control sample in rural area in Yanqing. The DHA is considered a direct measure of soil microbial activities because only the viable cells contain the DHA. The DHA activity affects the rate of soil nutrient availability for plants [24]. The lower DHA activity in urban soils indicates the lower rate of soil respiration, which suggests less microbial activity. CAT is responsible for protecting the cells from the oxidative damage [25]. The lower CAT activity of urban soil indicates the lower tolerance of oxidative stress. Also the catalase activity is significantly related to the microbial biomass [26]. The microbial biomass is considered as a temporal fertilizer storage tank that slowly releases the nutrients for plants. The lower CAT activity of urban soil of Beijing indicates the lower soil fertility along the roads in urban areas of Beijing. The urease enzyme plays an important role in the nitrogen and carbon cycles [24]. The URE catalyzes the hydrolysis of urea to carbon dioxide and ammonia. URE activity is positively correlated with total nitrogen [26]. The low URE activity of the urban soils of Beijing indicated the lower total nitrogen stored in the soil. While DHA and CAT activities were negatively correlated with increasing PM exposure, urban soil Urease activities increased with increasing PM exposure. Much of the urease activity in soil is also due to extracellular enzyme [27]. Lower activities of DHA and CAT both as intracellular enzymes associated with microbial metabolism, suggest less microbial activity. Less microbial activity would slow the breakdown of soil organic matter including enzyme proteins. Extracellular enzymes (including urease) could accumulate and stabilize, causing an apparent increase in URE with PM.

Overall, our data showed the degradation of soil quality along the roads in urban areas of Beijing as compared to the rural areas of Beijing. The soil enzymes play an essential role in the carbon, nitrogen, and phosphorus cycle. Lower soil enzyme activities might reduce the decomposition rate of organic matter and the availability of nutrient availability to plants in the soil along the major roads in urban areas of Beijing. The shortage in nutrient supply can adversely affect the growth of plants. The roots of plants suffering from malnutrition may not be able to grow as big and fast as those of healthy plants and release the same content of active enzymes into the soil. From the long-term perspective, the soil enzyme activities will show a continually decreasing trend along major roads in urban areas of Beijing.

We propose a hypothetical pathway model to interpret the impact of traffic-related air pollutions on soil enzyme activities. The first pathway is the wet deposition of heavy metals, which directly influences the soil enzymes. The heavy metals in the particular matter (e.g., Cd, Pb, Cu, and Zn) are deposited over the soil surface through the rainfall and runoff [28]. Correlation of the Cd, Pb, Cu, and Zn concentrations in particular matter and soil surface was 0.9, which can prove the transport of heavy metal from the air to the soil [29]. The average concentration of Cd, Pb, Cu, and Zn in urban soils of Beijing was found to be 0.215 mg/kg, 35.4 mg/kg, 29.7 mg/kg and 92.1 mg/kg. In contrast, the background value of Cd, Pb, Cu, and Zn is 0.119 mg/kg, 24.6 mg/kg, 18.7 mg/kg and 57. 5 mg/kg [30]. The concentrations of these heavy metals are significantly higher than the background values of Beijing. The toxicity of these heavy metals varies across difference soil enzymes [31]. Cd can inhibit dehydrogenase, catalase and urease while Zn can only inhibit catalase and urease [32]. Cu shows the negative inhibitory effect on dehydrogenase and urease [33]. Pb is less inhibitory to catalase and urease than Cd and Zn [32]. The synergistic inhibitory effect of Cd, Zn, and Pb is observed, which causes more reduction of enzyme activities than by the metals alone [32]. The toxicity of heavy metals is also influenced by the soil property (e.g., moisture, pH, organic matter) [33]. The full understanding of the impact of heavy metals on soil quality along the roadside needs a systematic investigation in the future. In the present study we found no significant differences in physical and chemical properties between rural and urban areas. Changes in urban and rural soil enzyme activities were not influenced by soil physical-chemical properties. The second pathway is the damage of plants caused by the exposure to the traffic pollutants. Two of major substrates for soil enzymes are plant roots and residues. Traffic pollutants (waste heat, CO, SO_2_, NO_x_, and VOC_s_) may affect plant health and growth [34,35]. The impaired plants may not have a comparative capability to release enzymes as the healthy plants do. From the long-term perspective, the accumulation and stabilization of soil enzymes is reduced in the roadside soil inurbane areas of Beijing.

Future studies need to verify the proposed pathways and uncover other unknown pathways of how traffic-related air pollution affects the soil enzymes and soil quality. The contribution of each pathway should also be quantified to help investigate the cost-effective strategies of mitigating the impact of traffic-related air pollution.

In this study, we employed soil enzyme activities as indicators of soil quality. However, there are arguments on the reliability of soil enzyme activities as soil quality indicators. The impact of pollutants on soil enzyme activities is variable depending on the soil type and pollutant type. For example, DHA activity is high in soil polluted with pulp and low in soil polluted with fly ash [36]. In this study, pollutants are consistent across the study areas, which mainly come from vehicle emissions. In the future, we should consider the pollutant types when comparing the soil quality near different land uses (residential, commercial, and industrial). Another limitation in using soil enzyme activities is the inability to discriminate between the effect of the pollutant and any prior degradation of the sites, or discriminate different pollutants. There are also other biochemical indicators available such as total nitrogen, total carbon and carbon/nitrogen for soil quality. The evaluation of soil quality degradation requires the information on soil enzyme activities as well as other soil biochemical indicators. Complex expression which combines the soil enzyme activities and other soil biochemical indicators could be a possible and feasible solution for soil quality assessment [36].

The degradation of soil quality is a great challenge to green space management along the traffic corridors in urban areas. To overcome the degradation of soil quality, the cultivation of green spaces along the traffic corridors in urban areas of Beijing needs to address the augmentation of microbial activities, plant selections, and traffic pollution control. Fertilization treatment by adding nitrogen and phosphorus is one engineering solution to increase microbe activities [37]. Fertilizers with different compositions can have differential impacts on different enzymes. The selection of fertilizers for the roadside soils in urban areas of Beijing needs proper investigations to facilitate the growth of specific plants. Coexistence of plants can also contribute to the increase of microbe activities and the improvement of soil quality [38]. In Beijing and other northern Chinese cities, Buxus megistophylla is widely used as the primary plant for road greening. Combination of plants mainly considers the aesthetic appeal of the roadsides. In the future, the proper combination of plants should also consider their effectiveness in increasing microbe activities and improving soil quality. The control of traffic-related air pollution is also required to improve the soil quality. Possible strategies include the filtration of metal particles and the replacement of the gasoline vehicles with clean vehicles powered by electricity from renewable energy sources [39]. The adoption of the particle filtration system and clean vehicles can significantly reduce the amount of toxic exhaust emissions.

Finally, we admit that this paper is a preliminary study on managing ecosystem services to cities by understanding the soil quality in greenspaces. We did not have a large observation dataset that should include multiple rural samples and long-term exposure records. The statistical analysis in the study cannot provide strong evidence for the impact of traffic-related air pollution on soil quality. In spite of the existence of weakness, the paper is intended to call for the attention to the tipping point of soil quality when investing and maintaining green spaces. If we fail to maintain a proper soil quality after greening, green spaces not only cannot help mitigate the impact of human activities, but also can lose their vitality under the pressure of human activities.

## 5. Conclusions

In this study, we assessed the impact of traffic pollution on soil enzymes along the roadsides in urban and rural areas of Beijing. We employed soil enzyme activities as indicators of soil quality. Results show the significant decrease in dehydrogenase, catalase activities along the major roadside in urban areas of Beijing.

DHA and CAT activities were negatively correlated with the increasing traffic pollution. In urban samples, URE activity was stimulated by the increasing of traffic air pollutants. Our findings showed that change in enzyme activities was not influenced by soil physical-chemical properties but by the higher level of traffic-related air pollutants.

The significantly lower enzyme activities in urban soils in comparison to rural soils indicate less microbial activity and the degradation of soil quality. The degradation of soil quality along the major roadsides imposes a great challenge on traffic corridor green space development. The potential strategies include the augmentation of microbe activities, plant selections, and traffic pollution control.
